# Autoantibodies during infectious diseases: Lessons from malaria applied to COVID-19 and other infections

**DOI:** 10.3389/fimmu.2022.938011

**Published:** 2022-09-15

**Authors:** Juan Rivera-Correa, Ana Rodriguez

**Affiliations:** ^1^ Biological Sciences Department, New York City College of Technology, City University of New York, Brooklyn, NY, United States; ^2^ Department of Microbiology, New York University School of Medicine, New York, NY, United States

**Keywords:** autoantibodies, malaria, atypical B cells, COVID-19, autoimmunity

## Abstract

Autoimmunity is a common phenomenon reported in many globally relevant infections, including malaria and COVID-19. These and other highly inflammatory diseases have been associated with the presence of autoantibodies. The role that these autoantibodies play during infection has been an emerging topic of interest. The vast numbers of studies reporting a range of autoantibodies targeting cellular antigens, such as dsDNA and lipids, but also immune molecules, such as cytokines, during malaria, COVID-19 and other infections, underscore the importance that autoimmunity can play during infection. During both malaria and COVID-19, the presence of autoantibodies has been correlated with associated pathologies such as malarial anemia and severe COVID-19. Additionally, high levels of Atypical/Autoimmune B cells (ABCs and atypical B cells) have been observed in both diseases. The growing literature of autoimmune B cells, age-associated B cells and atypical B cells in Systemic Lupus erythematosus (SLE) and other autoimmune disorders has identified recent mechanistic and cellular targets that could explain the development of autoantibodies during infection. These new findings establish a link between immune responses during infection and autoimmune disorders, highlighting shared mechanistic insights. In this review, we focus on the recent evidence of autoantibody generation during malaria and other infectious diseases and their potential pathological role, exploring possible mechanisms that may explain the development of autoimmunity during infections.

## Autoantibodies during infection

Autoantibodies are well-known mediators of pathology in autoimmune disorders, such as Systemic Lupus erythematosus (SLE) where they cause organ inflammation and damage (1). However, it has been less appreciated that autoimmunity is a common phenomenon during infections, including viral, bacterial and parasitic diseases such as HIV, tuberculosis or malaria, among others (2, 3). Many human infections with a highly inflammatory component, such as malaria and COVID-19, have been associated with high levels of autoantibodies targeting various host molecules, such nucleic acids (DNA, RNA), membrane proteins, carbohydrates, and phospholipids (such as phosphatidylserine (PS) (2, 3). Although infection-induced autoantibodies have been reported for a long time, only recently a pathogenic role during infection has been identified. For example, infection-induced autoantibody production has been associated with transient or post-infection pathologies, such as malarial anemia (4–8) Intriguingly, most of these autoantibodies are present at higher levels during acutely infected patients and drop upon treatment or resolution of infection (2). The regulation and roles of these autoantibodies are not completely understood.

ABCs and atypical B cells are known to expand and secrete autoantibodies in autoimmune disorders, such as SLE (9, 10). ABCs were largely described in mouse studies to accumulate with age, in autoimmune disease models and during acute viral infections and were delineated by expression of the non-classical B cell markers CD11c and T-bet (10). On the other hand, atypical B cells were initially described as a memory population that accumulate in patients with HIV, tuberculosis, repeated malaria exposures and other infections, as well as in autoimmune patients, and were mainly characterized by the lack of expression of CD27 and CD21, but also by expression of CD11c and T-bet (11, 12). During malaria, these atypical B cells have been implicated in secreting autoimmune antibodies against PS, and in contributing to malarial anemia (13, 14). ABCs and atypical B cells represent a heterogonous population with many different development origins, that could include both classical and non-classical routes, such as the extrafollicular (EF) route which has been associated with autoantibody secretion. Polyclonal activation and relaxation of B cell tolerance during complex infectious diseases, could account for this autoimmune phenomenon (15–17). Due to the variable nomenclature used in the literature to describe these cells in different settings, we will use “ABCs and atypical B cells” as a generalized term to describe Atypical/Autoimmune/Age associated B cells and Double Negative cells (DNs). Recently, ABCs and atypical B cells have been implicated in the autoimmune phenomena during infection, as they were shown to expand and secrete autoantibodies in autoimmune disorders, such as SLE. Concurrently, ABCs and atypical B cells have also been reported to expand in acutely infected patients with malaria (7, 8, 18), HIV ( (11, 19)), COVID-19 ( (20–23)) and other infections, as well as to accumulate in malaria-experienced individuals with repeated seasonal exposure (Portugal et al., 2017).In this review, we will summarize the evidence reinforcing the relationship between autoantibodies and pathology as well as possible mechanisms explaining its appearance. We will focus on malaria patients but will also expand to recent evidence in COVID-19 patients and other infections.

## Pathological role of autoantibodies during malarial anemia and other complications

Autoantibodies during both human and mouse malaria have been reported for decades, but until recently they had not been functionally studied. Malaria leads to production of a range of autoantibodies targeting all kinds of host molecules and cells, such as the phospholipid PS (2, 24, 25). Autoantibodies targeting PS have been directly shown to promote malarial anemia in rodent infections with *P. yoelii* through binding to PS on uninfected red blood cells (RBCs) and promoting their premature clearance (4). In this mouse model, young RBCs (reticulocytes) were preferentially targeted by these anti-PS antibodies, prolonging anemia recovery. These findings were validated in *P. falciparum*-infected patients which showed an inverse correlation between anti-PS antibodies and hemoglobin levels in different cohorts, including French travelers with post malarial anemia (4), acutely infected German travelers (7) and Ugandan pediatric patients with complicated *P. falciparum* malaria (6). Additionally, anti-PS antibodies were also corelated with anemia in a cohort of Colombian patients suffering from either *P. falciparum* or *P. vivax* malaria (Rivera-Correa et al., 2020), highlighting the presence of this correlation in different cohorts around the world. Lastly, these results were also expanded to four of the human *Plasmodium* species as evidenced by a study in Malaysia showing significant levels of anti-PS antibodies in *P. vivax*, *P. falciparum*, *P. malariae* and *P. knowlesi* malaria (5). In this cohort, anti-PS were associated with early anemia in *P. vivax* and *P. falciparum-*infected patients. In addition to anti-PS antibodies, *P. vivax*-malarial anemia has also been correlated with other autoantibodies targeting RBC surface proteins such as spectrin and band 3 in a Brazilian cohort (26, 27). Altogether, these studies show the strong relationship of anti-PS and other autoantibodies with malarial anemia in different human malaria cohorts around the world.

In addition to anemia, autoantibodies have been hypothesized to promote other malaria-associated pathologies (25). In a study of a pediatric Ugandan cohort suffering from severe *P. falciparum* malaria, anti-PS antibodies and anti-dsDNA antibodies were associated with acute kidney-injury (AKI), post-discharge mortality and morbidity, in addition to anemia (6). The mechanism involved in promoting other malaria-associated pathologies is not well understood. Previous studies on mouse models of malaria have linked dsDNA autoantibodies in promoting kidney pathology through the accumulation of immune complexes (28). Despite multiple *Plasmodium* species being reported to lead to kidney pathology, the relationship between kidney immune complex deposition and kidney pathology has only been reported in *P. malariae* malaria (29). This suggest that other non-autoantibody immune factors are needed in addition to promote kidney pathology in other human malarias underscoring the complexity of this phenomena (30). Lastly, there have also been several studies reporting autoantibodies against brain-associated antigens in patients suffering from *P. falciparum* cerebral malaria, but their pathogenic relevance remains to be determined (25). Although the pathogenic role of autoantibodies during malaria has been more evident, a possible protective role against infection cannot be disregarded. Specific autoantibodies have been correlated with protection against severe malaria (31, 32). Moreover, reports have shown that sera from patients with autoimmune diseases can bind to the parasite and inhibit parasite growth *in vitro* (33, 34). These findings suggest that autoantibodies could have divergent roles as both protective and pathogenic during malarial infection. These divergent roles have been comprehensibly reviewed in previous publications (2, 24, 25).

## Possible mechanisms leading to autoantibody production during malaria

The generation of autoimmunity involves a complex mix of genetic and environmental factors that are not well understood in autoimmune disorders and much less in infection. Epitope spreading, bystander activation, molecular mimicry, and cryptic epitopes are particular phenomena that explain the generation of specific autoantibodies during some infections, however, they do not seem to explain the broad variety of self-antigen targets observed in complex infections such as malaria (2) and COVID-19. To add to this complexity, malaria parasites are eukaryotic parasites that share many similar antigenic targets, such as PS and dsDNA, that could target both the parasite and the host cells equally. Additionally, hemolysis during malaria and other infections, exposes many host antigens, such as PS, that can activate the immune system and activate polyclonal B cell responses (35).

Autoantibodies are secreted by autoreactive B cells that get activated by a combination of specific signals that do not normally occur in non-pathogenic immune responses. A body of research, mainly in the SLE field, has described a specific B cell subset that is expanded and is able to secrete autoantibodies during autoimmune disorders called Age/Autoimmune B cells (ABCs and atypical B cells) in mice and a similar population called Double Negative B cells (DNs) in humans (9, 17). A similar population has been reported to be expanded during malaria (11) and in highly inflammatory infections such as HIV, tuberculosis and COVID-19. These cells differ from other B cell populations in the expression of characteristic markers such as low classical memory B cell markers CD21 and CD27, high expression of transcription factor T-bet and integrin CD11c as well as surface expression of FcRL5. Additionally, these cells have been reported to express chemokine receptors such as CXCR3 and other markers. A summary of the nomenclature and markers described for this population has been summarized in previous reviews (36, 37). Further evidence that ABCs and atypical B cells generated during infection and autoimmune disorders are closely related comes from scRNAseq analysis of these cells in malaria patients, which share similar transcriptional profiles with HIV patients, but also with patients from different autoimmune disorders (SLE, rheumatoid arthritis or common variable immunodeficiency), suggesting they share common drivers of expansion and function (38).

During malaria, studies have reported the refractoriness of human atypical B cells to secrete antibodies *in vitro* and reduced B cell receptor (BCR) signaling in response to soluble antigens (18), but atypical B cells were responsive to membrane-bound antigens (39). Additionally, indirect evidence implicates that atypical B cells secrete anti-malaria antibodies, suggesting divergent roles for these cells during malaria (36, 40).

Accordingly, a recent study reported the presence of malaria-specific atypical B cells and found that atypical B cells could secrete antibodies with T cell help (41). Lastly, atypical B cells proliferate in response to malaria, but also to vaccination, indicating that they are part of a wider alternative lineage of B cells that is a normal component of healthy immune responses (42). Additional studies have directly shown that ABCs and atypical B cells secrete autoantibodies (14). Accordingly, a report focused on studying the repertoire of ABCs and atypical B cells in malaria-experienced individuals revealed enrichment of VDJ gene usage associated inherently with autoreactivity (V_H_4-34) (38). Altogether these data suggest that ABCs and atypical B cells could be major secretors of pathogenic autoantibodies, such as anti-PS. These studies highlight the complexity of ABCs and atypical B cells and their possibly divergent roles in both protective and pathogenic responses during malaria (43).

Recent insights into the mechanisms leading to autoreactive B cell generation have been elucidated in different mouse models. In autoimmune settings, the integration of primarily three signals are needed to generate ABCs and atypical B cells: BCR signaling, specific type 1 cytokines (IL-21 and/or IFNγ) and nucleic acid sensing toll-like receptors (TLR7 and TLR9) (9, 10, 44). During malaria, the integration of BCR signaling, IFNγ and TLR9 were deemed essential for ABCs and atypical B cells expansion and anti-PS autoimmunity during mouse *P. yoelii* infection (13). Similar findings were published in *P. falciparum* malaria patients, describing how IFNγ and TLRs were important for ABCs and atypical B cells generation (11, 12, 45). A recent study also expanded the role of IFNγ in promoting an ABCs/Atypical B cell phenotype in *P. vivax* patients (46). In the SLE field, the integration of these cytokines and TLRs, have been attributed to promote an alternative B cell differentiation pathway titled the extrafollicular (EF) pathway (47). In contrast to the classical germinal center (GC) pathway that gives rise to long-lived memory B cells or plasma cells, the EF route through polyclonal activation gives rise to short-lived plasmablasts/plasma cells (PB/PCs) (16, 17). The EF route is considered the main route by which DNs (analogs of ABCs and atypical B cells, named for being CD27^-^IgD^-^) can arise and secrete autoantibodies in SLE patients. Polyclonal B cell activation has been a hallmark during malaria and contributes to the B cells dysfunction observed during the disease (48). Accordingly, a study on mouse *P. yoelii* malaria, revealed that hemolysis-induced PS polyclonal activation of B cells, through PS-receptor AXL, accounted for great part of the polyclonal responses that lead to the accumulation of short lived PB/PCs that secrete non-specific antibodies and limit protective humoral immunity (35). In this study, they also reported that blocking PS exposure limits non-specific polyclonal PB/PC expansion and reduces *P. yoelii* infection in mice. Moreover, this accumulation of short-lived PB/PCs dampens the essential GC response needed for proper anti-malarial antibody responses (49). Additionally, the extension of time by which this polyclonal activation of B cells is prolonged could account for reports of sustained autoantibodies post-infection (for at least 1 month in *P. vivax* and *P. falciparum* infections) that may contribute to associated pathologies, such as post-malarial anemia and increased hospital post-discharge mortality (4, 24) (6). Altogether, these data suggest that these mechanisms, that are possibly shared between SLE and malaria, could explain the activation of the pathogenic autoantibody responses we see during acute malarial infection.

## Lessons from malaria applied to COVID-19 and other infections

Other highly inflammatory infections have been associated with generation of autoantibodies, most recently noted in the ongoing COVID-19 pandemic (3, 50). Similar to malaria, high levels of circulating autoantibodies have been reported in COVID-19 patients, but surprisingly, circulating immune complexes were not increased in these patients. The autoantibodies include similar targets to malaria, such as PS and dsDNA (51), but also expand to newer targets such Annexin A2 (52), that have not been studied in malaria patients. The autoantibody repertoire seems to be broad during COVID-19 and is not clear if it’s selected preferably against any autoantigen although their pathogenic role has been highly suggested by multiple studies (53, 54). The malaria-shared autoantibodies anti-PS and anti-dsDNA have been correlated with severity in COVID-19-patients (51), similarly as they have in malaria (**Table 1**). Autoimmune anti-Annexin A2 also correlated with mortality (52). A study in mice, reported that a range of anti-phospholipid antibodies could promote the pathological coagulation defects highly associated with COVID-19 infections (55). Moreover, the number of antibodies found targeting immune molecules such as cytokines (ex. Type I interferon) has been a highly reported phenomenon in severe COVID-19 (57–59). Accordingly, malaria has also been associated with targeting of immune components such as *IFNGR2* (60), which could distinguish malaria from bacterial blood infection in a small cohort of Ghanese children. Furthermore, early detection of a set of autoantibodies that included anti-IFN-a2, and five anti-nuclear autoantibodies (ANAs) (Ro/SS-A, La/SS-B, U1-snRNP, Jo-1, and P1) that are also commonly associated with (SLE), could anticipate distinct patterns of the puzzling phenomenon of Post-acute sequelae of COVID-19 (PASC) or “Long-COVID” (56). These reports suggest pathological implications of a range of autoantibodies in COVID-19 patients both at acute and post-infection manifestations. The mechanisms that give rise to autoantibodies during COVID-19 could be similar to the ones in malaria and SLE patients (61). Various studies have reported the expansion of ABCs and atypical B cells (20, 62), a phenomenon that could be explained by the enhanced EF route of B cells reported in COVID-19 patients (63). Additionally, the “relaxation” of B cell tolerance has been suggested as an additional mechanism promoting autoreactive B cells and autoantibody secretion in COVID-19 patients (64).

**Table 1:** Autoantigen targets for autoantibodies and their associated pathology during malaria or COVID-19. n/d, not determined.

Self-antigen	Malaria	Associated with Atypicals or ABCs?	COVID-19	References
**Phosphatidylserine (PS) and/or other Phospholipids(PL)**	Anemia (*P. falciparum and P. vivax*) Acute kidney injury (AKI) (*P. falciparum)* Mortality and morbidity (*P. falciparum)* Complicated malaria (*P. vivax)*	Yes	Severity Coagulation defects (general αPL)	(4–8, 51, 55)
**Double stranded-DNA (dsDNA)**	Anemia (*P. falciparum)* Acute kidney injury (AKI) (*P. falciparum)*	Yes	Severity	(6, 7, 51)
**Red Blood Cell whole lysates or specific protein antigens** **(ex. Band 3 and Spectrin)**	Anemia (*P. falciparum* and *P. vivax)* Complicated malaria (*P. vivax)*	Yes	Severity	(6–8, 25, 26)
**Annexin A2**	n/d	n/d	Severity Mortality	(52)
**Other autoantibodies**	Cerebral malaria	n/d	Severity Mortality Long-COVID	(3, 25, 53, 54, 56)

Many other infections also lead to similar autoantibodies as observed during malaria (2). Different anti-phospholipid antibodies have been reported during infections of important global pathogens such as *Mycobacterium tuberculosis* (65), HIV (66) and others such as hepatitis C, cytomegalovirus, varicella zoster, Epstein-Barr virus, adenovirus, and parvovirus B (67, 68). Active tuberculosis has been associated with a range of autoantibodies including with high levels of anti-cardiolipin and other anti-phospholipid antibodies (65, 69). Additionally, autoantibodies targeting RBC components have been reported to increase tuberculosis susceptibility in HIV patients through erythrophagocytosis (70), suggesting a pathogenic role for autoantibodies in co-infection scenarios. Furthermore, anti-phospholipid antibodies, such as anti-cardiolipin, have been utilized as a diagnostic tool for active *Treponema pallidum* infections, being one of the primary methods to diagnose syphilis in humans (71). A similar application was recently suggested for Lyme disease diagnosis (72). Anti-phospholipid and anti-ganglioside autoantibodies were also reported to be correlated in Zika virus-associated Guillain-Barré syndrome patients from Brazil (73, 74). Moreover, autoimmunity against PS and ABCs and Atypical B cell expansion was reported to delay anemia recovery in mice infected with African trypanosome *Trypanosoma brucei* (75). These findings were translated to a cohort of Ugandan Human African trypanosomiasis (HAT) patients where anti-PS levels were elevated in acutely infected patients. Similarly to COVID-19 and malaria, cytokines can be autoantibody targets during different infections such as: type II IFN during infections with intra-macrophagic microbes, IL-17A/F during mucocutaneous candidiasis and IL-6 during staphylococcal diseases (76). Since the signals leading to ABCs/Atypical B cell expansion, such as TLR ligation and IFN-γ, are present in many other infections, these cells are proposed to be a possible common source for autoantibodies during different infections (11, 15). Altogether, these data suggest a global pathogenic role of autoantibodies during malaria, COVID-19 and other infections with shared auto-antigen targets and related mechanisms of pathogenesis.

## Conclusions

High levels of autoantibodies are observed in many relevant infections, such as malaria and COVID-19. Their presence during infection has been reported extensively, but their contribution to pathology has been a recent research focus. The growing literature on the activation and expansion of ABCs and atypical B cells in autoimmune disorders such as SLE, has contributed with mechanistic insights that may be relevant for the generation of autoantibodies during malaria, COVID-19 and other infections.

**Figure 1:**
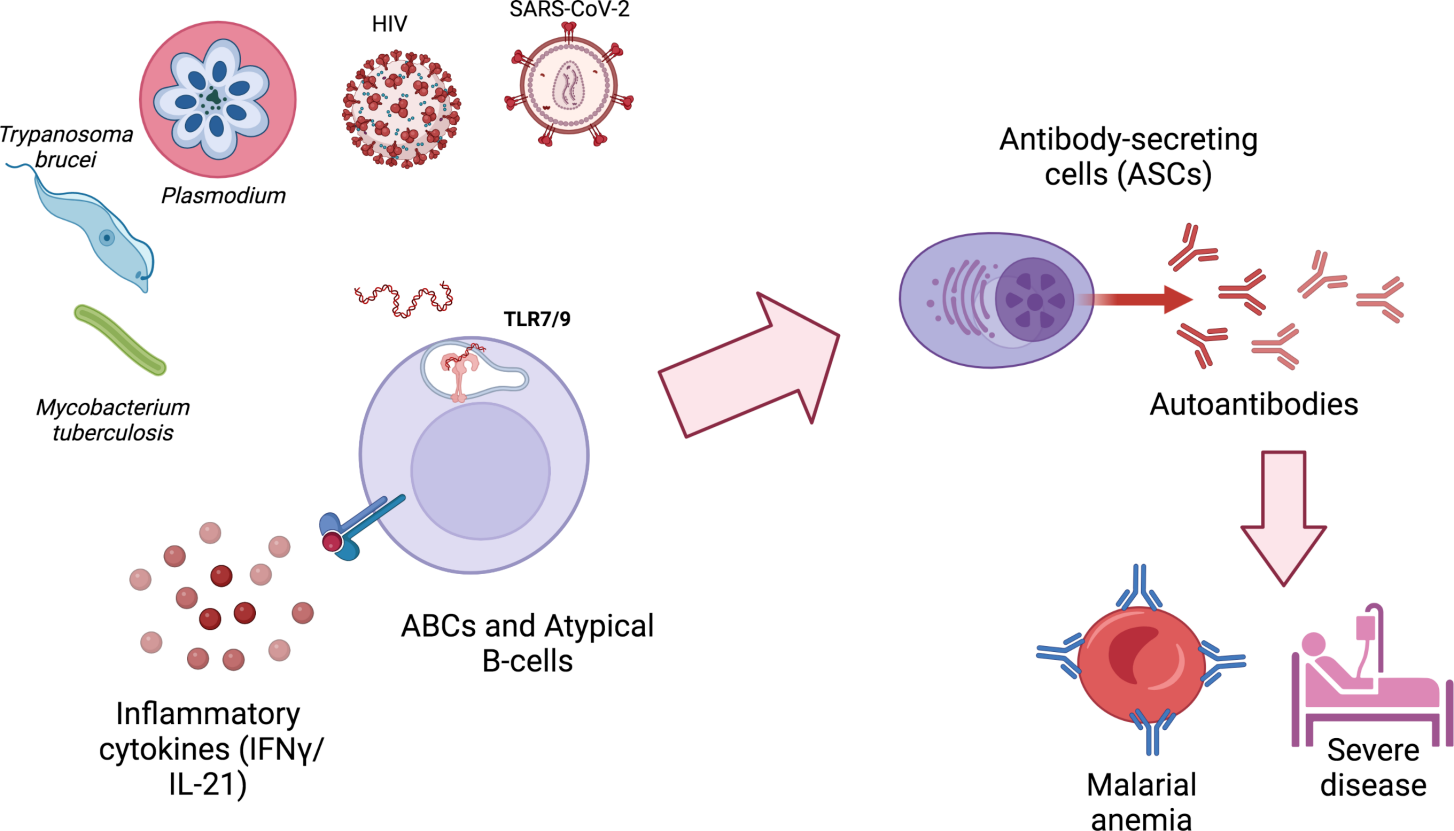
ABCs and atypical B cells are generated during different infections and secrete autoimmune antibodies that can contribute to pathology. ABCs and atypical B cells generation during infections requires at least two signals: 1) the activation of TLR9 or TLR7, typically by nucleic acids derived from the infectious agent or by host mitochondrial DNA released by neutrophil extracellular traps (NETs); 2) exposure to IFN-γ and/or IL-21. Autoimmune antibodies generated by autoimmune antibody-secreting cells (ASCs) can contribute to pathology and severe disease. Created with BioRender.com.

Specifically in malaria, autoantibodies contribute to pathogenesis through the binding of anti-PS autoantibodies to uninfected erythrocytes, promoting malarial anemia. The expansion of ABCs and atypical B cells and their ability to secrete anti-PS and other autoantibodies in both mouse and human malaria marks them as a primary candidate responsible for the generation of autoimmunity during infection. The signals driving ABCs and Atypical B cell expansion, such as TLR ligation, are present in many autoimmune disorders as well as infections, suggesting shared mechanistic pathways for autoreactivity in scenarios as diverse as SLE, malaria and COVID-19 (**Figure 1**). However, the transient aspect of infection-induced autoantibodies and the heterogeneity of ABCs and atypical B cells and their divergent functional roles during malaria highlight the complexity of this phenomenon during infection. Further studies are needed to understand the mechanisms by which these cells arise, explore fully their different roles and explain their dynamics during malaria and other infections.

## Author contributions

All listed authors have made a substantial, direct and intellectual contribution to the work, and approved it for publication.

## Funding

AR was supported by NIH grant R21AI151349.

## Conflict of interest

The authors declare that the research was conducted in the absence of any commercial or financial relationships that could be construed as a potential conflict of interest.

## Publisher’s note

All claims expressed in this article are solely those of the authors and do not necessarily represent those of their affiliated organizations, or those of the publisher, the editors and the reviewers. Any product that may be evaluated in this article, or claim that may be made by its manufacturer, is not guaranteed or endorsed by the publisher.
